# Improving Blood Pressure Screening in Neonatal Follow-up Clinic: A Quality Improvement Initiative

**DOI:** 10.1097/pq9.0000000000000559

**Published:** 2022-06-14

**Authors:** Rachel S. Flynn, Jacqueline Zedalis, Michelle R. Denburg, Judy C. Bernbaum, Sara B. DeMauro

**Affiliations:** From the * Division of Neonatology, Lehigh Valley Health Network, Allentown, Pa; † Division of Neonatology, Children’s Hospital of Philadelphia, Pa; ‡ Hospital of the University of Pennsylvania, Philadelphia, Pa; § Division of Nephrology, Children’s Hospital of Philadelphia, Philadelphia, Pa.

## Abstract

**Introduction:**

The American Academy of Pediatrics recommends blood pressure screening at every health care encounter in children younger than 3 years if they have a history of prematurity or other neonatal complications requiring intensive care because these children have an increased risk for hypertension.

**Methods:**

A multidisciplinary team conducted a quality improvement initiative to improve blood pressure screening at a single-center outpatient neonatal follow-up clinic. We developed a focused intervention program including a standardized blood pressure measurement protocol, staff training and education, and streamlined documentation. We conducted two Plan-Do-Study-Act cycles from November 2019 to January 2021. The outcome measure was the percentage of patients with a blood pressure measurement. Process measures included the percentage of medical assistants educated on the new protocol, percentage of patients 3 years, and younger old with the first blood pressure measurement taken from the right arm, and the percentage of patients 1 year and younger with 3 documented blood pressures. The balancing measure was staff satisfaction with time to obtain vital signs. We used statistical process control charts and Wilcoxon rank-sum test.

**Results:**

At baseline, only 15.3% of patients had documented blood pressure. During the 10-month intervention period, there were 954 patient visits. Overall, blood pressure measurement increased to 54.7% with study interventions. The balancing measure was not negatively impacted.

**Conclusions:**

After implementing a program of focused interventions, we substantially improved the frequency of blood pressure measurements and increased adherence to American Academy of Pediatrics screening guidelines. Improved blood pressure screening allows us to identify and evaluate at-risk infants after hospital discharge.

## INTRODUCTION

Although uncommon in healthy full-term infants, hypertension can occur in up to 3% of neonates admitted to the neonatal intensive care unit (NICU).^1,2^ Infants with a history of NICU hospitalization remain at increased risk for hypertension, which may be detected well after discharge.^1,3^ Preterm birth, low birth weight, bronchopulmonary dysplasia, intraventricular hemorrhage, acute kidney injury, patent ductus arteriosus, and history of umbilical catheterization are risk factors for hypertension.^4^ Preterm birth and low birth weight are associated with hypertension and coronary heart disease in adults.^5^ The higher incidence and delayed appearance of hypertension support routine blood pressure monitoring following NICU discharge.

Given the vital importance of early detection of hypertension, the American Academy of Pediatrics (AAP) recommends measuring blood pressure at routine well-child visits.^6^ The 2004 National High Blood Pressure Education Fourth Report recommended a blood pressure measurement at every health care encounter in children younger than 3 years if they have a history of prematurity or other neonatal complications requiring intensive care.^7^ The updated AAP guidelines in 2017 reiterated these recommendations.^6^ Despite clinical guidelines recommending blood pressure screening at every health care encounter for NICU survivors under 3 years of age, a significant gap exists between guidelines and practice. One study has assessed adherence to AAP clinical practice guidelines within a large pediatric healthcare system.^8^ Among children younger than 3 years of age with risk factors for hypertension, only 38% had at least 1 blood pressure documented. Only 4% of primary care encounters had blood pressure documented.

There was no standard approach to blood pressure screening in the neonatal follow-up clinic at the Children’s Hospital of Philadelphia (CHOP). However, studies in adults suggest multi-pronged interventions including staff education, clinical training, automated devices, and support systems for change may successfully improve blood pressure measurement in primary care clinics.^9,10^ Here, we describe the development of interventions to improve the low rate of blood pressure screening in the CHOP neonatal follow-up clinic using the Model for Improvement framework. The objective of this project was to increase the percentage of patients in the CHOP neonatal follow-up clinic who have a blood pressure measurement taken from 15% to 35% over 6 months.

## METHODS

### Context

The neonatal follow-up clinic at the Buerger Center for Advanced Pediatric Care provides comprehensive medical and developmental care for premature and medically complex infants after they leave the hospital. The majority of patients seen after discharge are from the Hospital of the University of Pennsylvania Intensive Care Nursery and the Children’s Hospital of Philadelphia (CHOP) neonatal/infant intensive care unit. The University of Pennsylvania Intensive Care Nursery is a 38-bed, level III unit with approximately 700 annual admissions. The CHOP neonatal/infant intensive care unit is a quaternary, 98-bed unit with over 1,200 annual admissions. All infants born younger than 32 weeks gestation or weighing <1500 g at birth are referred to the neonatal follow-up clinic and any infant with neurologic, cardiorespiratory, or other significant medical concerns. More than 1,300 patient visits are completed in the follow-up clinic each year. The first visit occurs within 2 to 3 months after discharge, with the number and frequency of subsequent appointments depending on the patient’s individual medical and developmental needs.

The multidisciplinary project team included neonatologists, a pediatrician, a nephrologist, nurse practitioners, certified medical assistants, and a clinical quality improvement (QI) coordinator. The team created a protocol and program to improve the frequency of blood pressure screening in the neonatal follow-up clinic.

### Intervention

**Fig. 1.**
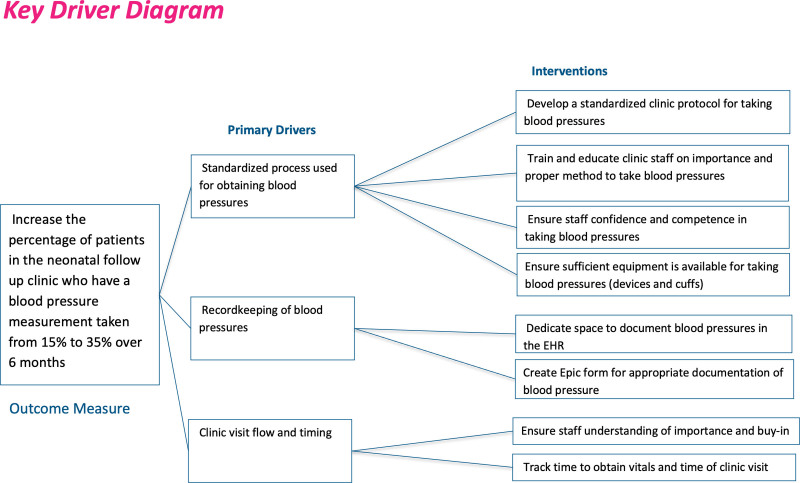
Key driver diagram detailing the outcome measure, drivers, and interventions for the project.

The team developed a key driver diagram (Fig. 1) using the Institute for Healthcare Improvement QI principles. We then developed focused interventions using key drivers, including the following components:

1. Developing a standardized clinic blood pressure protocol
2. Training and education for clinic staff on the new protocol
3. Streamlined medical record documentation

We implemented and evaluated interventions using sequential Plan-Do-Study-Act (PDSA) cycles.

**Fig. 2.**
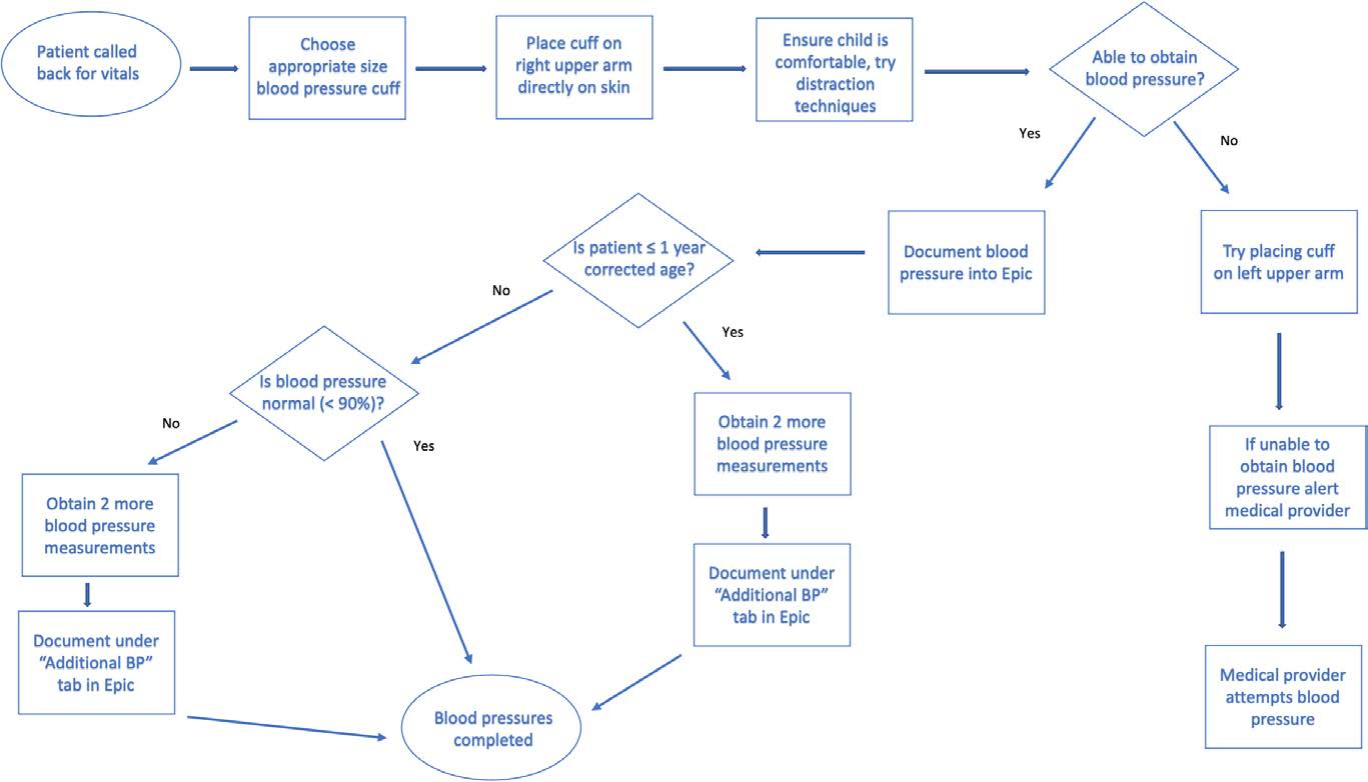
Process map detailing the standardized blood pressure measurement protocol for the follow-up clinic established by the team.

The team established a standardized blood pressure measurement protocol (see Materials and Methods, Supplemental Digital Content 1, http://links.lww.com/PQ9/A374) for the follow-up clinic and created a flowchart to detail the sequence of steps (Fig. 2). The clinic protocol directly incorporated AAP recommendations regarding measuring a blood pressure at every health care encounter in children younger than 3 years with a history of a NICU stay; use of an oscillometric device in infants and toddlers until they can cooperate with auscultatory BP; indications to repeat a blood pressure measurement; and blood pressure measurement technique.^6^ To minimize confusion and simplify steps for providers, the clinic protocol recommended obtaining a blood pressure measurement in every clinic patient, even if over the age of 3 years. Additionally, the clinic protocol suggested obtaining multiple blood pressure measurements in patients 1 year and younger corrected age to obtain more accurate readings.

Per the protocol, a certified medical assistant took a set of vitals after a patient checked in to the clinic. The appropriate size cuff was determined based on age using the color-coded guide and placed on the patient. The protocol emphasized placing the cuff directly on the right upper arm on the skin. The patient’s position was documented in the EHR. Ideally, 3 blood pressure measurements were obtained for any patient 1 year and younger corrected gestational age or if the patient was older than 1 year corrected gestational age with an elevated initial blood pressure measurement (≥90th percentile based on cutoffs from the fourth report) to get an average of the readings.^7^ If the medical assistant could not obtain a blood pressure reading, they alerted the medical provider (physician or nurse practitioner), who would then attempt to obtain a blood pressure measurement using an automated blood pressure measurement device (CarescapeV100; GE Healthcare, Milwaukee, WI).

Before protocol implementation, a project team member trained the clinic medical assistants on the process and best blood pressure measurement practices during group presentations and individual educational sessions to ensure understanding of the new workflow. Medical providers were educated with a presentation on the new protocol during a monthly clinic meeting before the first PDSA cycle. In addition, we posted educational infographics in provider workspaces. The infographics detailed the number of blood pressure measurements needed and the proper technique.

Vitals are documented and stored using an electronic health record (EHR; Epic Systems, Verona, WI). We adjusted the EHR workflow based on staff feedback and recommendations. Previously, the medical assistant or provider would need to create a “new reading” to document each additional set of vitals, which entailed additional clicks and data entry. In addition, we added a blood pressure tab to the navigator to streamline blood pressure documentation. This modification allowed providers to enter 2 additional blood pressures without creating new readings and allowed easier extraction of blood pressure data from the EHR.

We referred the patient to pediatric nephrology if the systolic blood pressure was ≥99th percentile in 2 out of 3 measurements. If the systolic blood pressure was ≥90th percentile but <99th percentile, the medical provider would communicate to the pediatrician through the EHR recommending repeat measurements in subsequent primary care or specialty care visits. During the neonatal follow-up visit, all recommendations were also discussed with the patients’ families.

### Study of the Intervention

Baseline data were collected from the EHR for 11 months before the PDSA cycles (January 2019 to November 2019). We collected intervention data for 10 months (November 2019 to January 2021), not including the months the clinic was closed due to the coronavirus disease 2019 (COVID-19) pandemic. The team lead, clinical QI coordinator, and data engineer created an EHR-based data report dashboard. The dashboard automatically extracted age and blood pressure measurement data, including blood pressure values, cuff location, and patient position from all patients seen in the follow-up clinic. The first PDSA cycle began in November 2019 with the new standardized clinic blood pressure protocol roll out after training the clinic staff and EHR updates. The second PDSA cycle began in June 2020 once the clinic reopened after being closed due to the COVID-19 pandemic from March 2020 to June 2020. We distributed a survey to measure provider satisfaction in October 2019 for a baseline and after 6 months of project implementation. All members of the clinic staff received the surveys via electronic communication.

### Measures

We obtained data for all measures through EHR chart abstraction. The primary outcome measure was the percentage of patients with a blood pressure measurement taken in the follow-up clinic. This measure was defined as the number of patients who had a blood pressure measurement taken divided by the number of patients seen in the clinic on a 2-week basis. The process measures were the percentage of medical assistants educated on the new blood pressure protocol, the percentage of patients 3 years and younger old with the first blood pressure measurement taken from the right arm, and the percentage of patients 1 year and younger corrected gestational age with 3 documented blood pressures. The percentage of patients 3 years and younger old with the first blood pressure measurement taken from the right arm was defined as the number of patients 3 years and younger old who had a blood pressure measurement taken in the right arm divided by the number of patients 3 years and younger old seen in clinic on a 2-week basis. The percentage of patients 1 year and younger corrected gestational age with 3 documented blood pressures was defined as the number of patients 1 year and younger corrected gestational age with 3 documented blood pressures divided by the number of patients 1 year and younger corrected gestational age seen in clinic on a 2-week basis. The balancing measure was staff satisfaction with time to obtain vital signs, as measured by a survey including the question “obtaining vital signs takes too little time, the right amount of time, or too much time?” On a sliding scale, staff was asked to what extent they felt obtaining vital signs took too little time (0), the right amount of time (50), or too much time (100).

### Analysis

We interpreted the primary outcome measure and 1 process measure with a p-chart for statistical process control, created using QIMacros software (KnowWare International, Denver, Colorado). Control limits were adjusted using traditional health care special cause variation rules. Eight consecutive data points above or below the mean centerline determined special cause variation.^11^ The balancing measure was compared by a Wilcoxon rank-sum test performed in Stata 16 (Stata Corp, College Station, TX) with *P* < 0.05 as the level for statistical significance.

### Ethical Considerations

This study was determined to be a QI initiative by the Children’s Hospital of Philadelphia Institutional Review Board and was exempt from further review.

## RESULTS

**Fig. 3.**
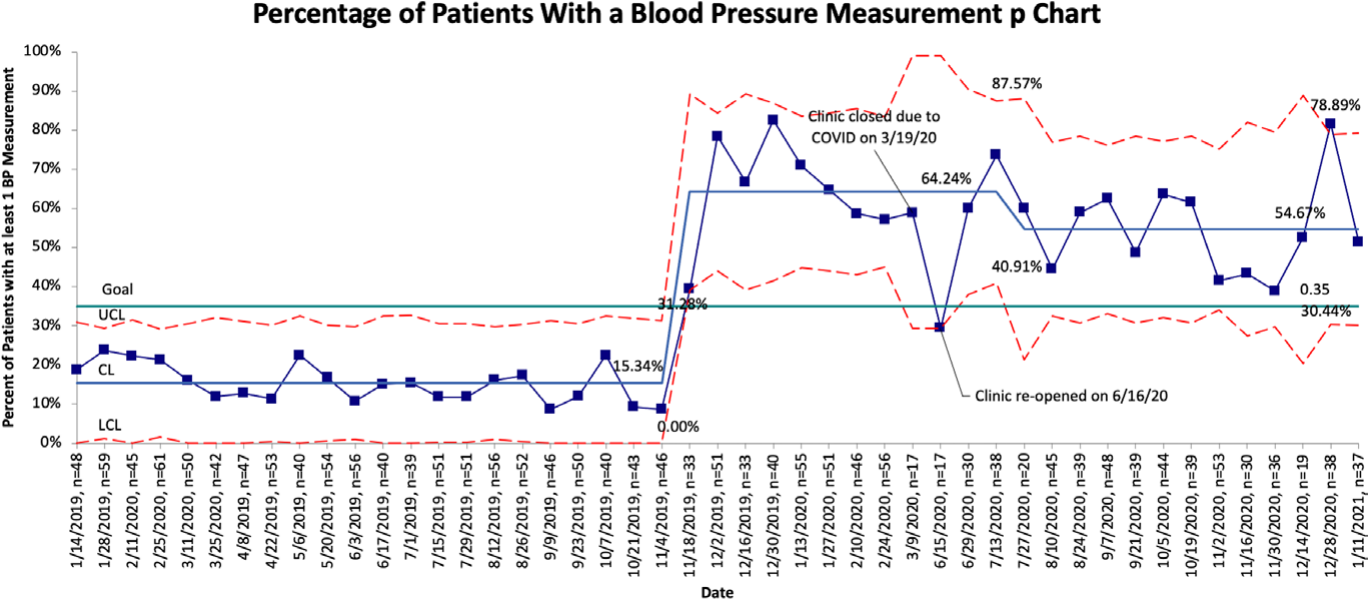
Control chart of the primary outcome, January 2019 to January 2021. LCL, lower control limit; UCL, upper control limit.

During the 11-month baseline period, there were 1,102 patient visits, and 15.3% of patients had a blood pressure measurement documented in the EHR. During the 10-month intervention period, there were 954 patient visits. The percentage of patients who had blood pressure taken increased to 64.2% during the first PDSA cycle and then 54.7% once the clinic reopened after being closed due to the COVID-19 pandemic. Overall, a control chart of the primary outcome data demonstrated a centerline shift from a baseline of 15.3% to 54.7% (Fig. 3).

The COVID-19 pandemic has had a deep impact on many aspects of health care delivery. Our clinic provided care via telemedicine during the 3 months we were prohibited from providing in-person care.^12^ Unfortunately, we were unable to obtain blood pressure measurements on our patients during that time. Upon clinic reopening, we adjusted the medical assistant staffing model. Before COVID-19, there were multiple medical assistants assigned to the clinic throughout the week; however, after reopening in June 2021, a single primary medical assistant from the previous group was assigned to the clinic, with occasional coverage by others when needed. After clinic reopening, there were no additional teaching sessions or reminders to adhere to the blood pressure protocol.

**Fig. 4.**
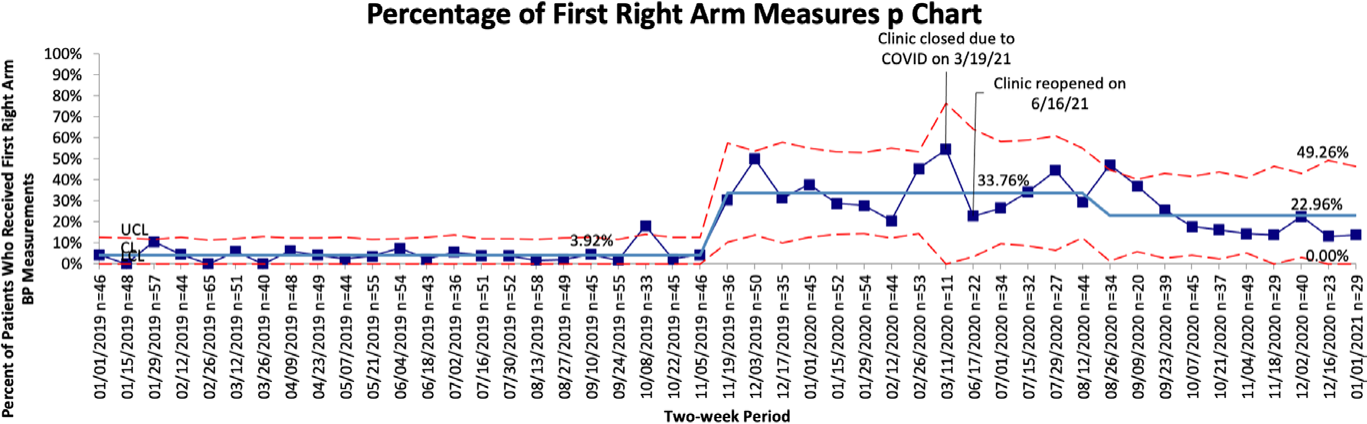
Control chart of the percentage of patients 3 years and younger old with the first blood pressure measurement taken from the right arm, January 2019 to January 2021. LCL, lower control limit; UCL, upper control limit.

We educated 100% of medical assistants on the new blood pressure protocol. The percentage of patients ≤3 with the first blood pressure measurement documented as taken from the right arm increased from a baseline of 3.9% before the first PDSA cycle to 23.0% (Fig. 4). The percentage of patients 1 year and younger corrected gestational age with 3 documented blood pressures did not increase from a baseline of 0%. However, there was an initial increase in the percentage of patients 1 year and younger corrected gestational age with 3 documented blood pressures during the first 4 months of the intervention period. This improvement was not sustained and did not meet the criteria for a baseline shift.

After 6 months of project implementation, the prestudy baseline survey and the follow-up survey had a response rate of 100% (10 staff members). In addition, the balancing measure of staff satisfaction with time to obtain vital signs were not negatively impacted by study interventions with a median score of 75 (interquartile range 66−80) preintervention versus a median score of 75 postintervention (interquartile range 75−88) and *P* = 0.49 when compared with the Wilcoxon rank-sum test. In free-text comments, staff most often noted the concern of falsely elevated blood pressure measurements due to issues such as patient agitation.

## DISCUSSION

This QI initiative demonstrated a significant improvement in blood pressure measurement in an outpatient neonatal follow-up clinic. The percentage of patients in the neonatal follow-up clinic who had a blood pressure measurement increased from 15.3% to 54.6% without a decrease in clinic staff satisfaction with the time required to obtain the vital signs. By developing a standardized protocol, additional staff training and education, and streamlined medical record documentation, the clinic improved adherence to AAP blood pressure screening guidelines.

Our neonatal follow-up clinic had a low baseline rate of blood pressure screening. This rate is similar to the low rates of blood pressure screening in patients less than 3 years of age with risk factors for hypertension documented in prior studies.^8,13^ A mixed-methods study of a pediatric practice-based research network found that blood pressure was only documented in 14% of health care visits in children younger than 3 years of age with a history of prematurity.^13^ Reasons for low adherence rates to the AAP blood pressure screening guidelines are postulated to include provider time constraints and lack of a standardized protocol for measuring blood pressure.

This initiative is the first documented in the literature to improve blood pressure screening in an outpatient neonatal follow-up clinic. There have been few reported efforts to improve blood pressure measurement in outpatient pediatric general or subspecialty practices, except pediatric renal transplant clinics. The Improving Renal Outcomes Collaborative used standard QI methodology and training to successfully improve and standardize blood pressure measurement in multiple renal transplant clinics.^14^ After engaging in online interactive workshops, each center developed specific interventions, including training clinic staff, providing appropriate supplies, and developing provider alerts. Over an active intervention period of 20 weeks, appropriate blood pressure measurement improved from 11% to 78%. In contrast, our project was a single-center study in a neonatal follow-up clinic rather than multiple pediatric kidney transplant centers. Our project emphasized blood pressure screening and included a longer intervention period.

By improving blood pressure measurement in children following discharge from the NICU, we aim to improve short- and long-term care of this vulnerable population at increased risk for hypertension. Elevated childhood blood pressure is associated with adult hypertension and metabolic syndrome.^6^ Measuring blood pressure over time using a standard technique allows us to recognize children who may require hypertension treatment and identify children at risk of developing adult cardiovascular disease.^15^ This QI initiative demonstrates that it is possible to improve practice with focused interventions.

Overall, the clinic achieved a robust implementation of the new protocol. The adherence was robust enough to sustain change even with disruptions and adjustments due to the COVID-19 pandemic. However, there was a drop in both the primary outcome measure and the process measure of right arm blood pressure measurement after the clinic reopened, which may have been due to a lack of reeducation or worry regarding COVID-19 precautions. The slight decline in blood pressure measurements from January to March 2020 may reflect a leveling off after the immediate rapid improvement secondary to the initiation of tailored interventions or a need for more regular reminders. Future strategies to improve protocol compliance might include regular protocol reeducation sessions, an EHR reminder, or an EHR mandatory record field. When compared with adult clinic efforts to improve documentation of blood pressure measurement, our team demonstrated similar improvements. For example, after implementing a mandatory computer field, the proportion of patients who had their blood pressure measured increased from 40.6% to 58.5% in 9 clinics in a preferred provider organization in Tel Aviv.^16^ There was imperfect adherence to protocol elements, as evidenced by the low but improved percentage of patients with the first blood pressure measurement taken from the right arm and poor adherence to obtaining 3 blood pressure measures in children 1 year and younger corrected gestational age.

Despite the strengths of this study, there were several limitations. We designed the interventions for use in a large outpatient neonatal follow-up clinic. Therefore, the results may not generalize to other clinics or hospital settings with different staffing models or access to equipment. Additionally, a key component of our intervention involved streamlined EHR documentation, which would not be possible in a clinic without an advanced EHR. Due to the nature of the clinic workflow, we were unable to assess time to obtain blood pressures, which would have been a useful metric. We used staff satisfaction as a proxy for time to obtain vitals instead. Finally, we have not yet studied how often providers make appropriate referrals for elevated blood pressures.

This initiative will be expanded to include other neonatal follow-up clinic sites within the CHOP Newborn Care Network. Further work includes evaluating how often appropriate referrals for elevated blood pressures were placed and assessing how frequently referred children were diagnosed with and treated for hypertension to determine whether this program improves patient care and outcomes.

## CONCLUSIONS

This study demonstrated that implementing a program of focused interventions can enhance adherence to blood pressure screening guidelines by improving blood pressure measurement in an outpatient follow-up clinic. Other clinics can apply this framework to improve blood pressure screening in children at risk of hypertension.

## DISCLOSURE

The authors have no financial interest to declare in relation to the content of this article.

## ACKNOWLEDGMENTS

The authors would like to acknowledge the team members at the Buerger Center for Advanced Pediatric Care neonatology follow-up clinic, without whom this project would not have been possible.

## Supplementary Material


