# Supplementary material for: Improving Blood Pressure Screening in Neonatal Follow-up Clinic: A Quality Improvement Initiative
Source: Pediatr Qual Saf. 2022 Jun 14;7(3):e559. doi: 10.1097/pq9.0000000000000559 (PMC9197357; doi:10.1097/pq9.0000000000000559)
Supplement: Supplementary file 1 [file pqs-7-e559-s001.pdf]

SDC, Materials and Methods

Blood Pressure Measurement Protocol for Neonatal Follow Up Clinic

Device Type: Oscillometric- Dinamap V100

Infant Position: Prone or supine
- -If needed infant can be in parent or guardian's lap for comfort

Infant Clothing: Blood pressure (BP) should be taken directly on arm and not over clothing

Cuff Size: Appropriate for neonates- cuff width to arm circumference ratio 0.45-0.7
- -Can use color coded cuffs
- -Ideally: measure arm circumference with a tape first midway between olecranon and the acromion with shoulder neutral and elbow flexed to 90 degrees
- -Width of the cuff should be around 40% the upper arm circumference. The length should encircle 80-100% of the upper arm.
- -When cuff sizes overlap for a specified arm circumference, choose the larger size cuff

Cuff Location: Right upper arm, if unable to obtain try left upper arm

Timing:
- -Infant should be asleep, or if awake should be kept quiet
- -Ensure that the infant is not disturbed for as long as possible after the cuff is placed
- -If unable to obtain BP readings, please alert physician, obtain cuff from 2nd machine and leave on infant

Number of Blood Pressure Readings: Ideally 3 readings, 2 minutes apart for <1 year of age, minimum of a single blood pressure reading for all patients
- - ≤ 1 year corrected gestational age
  - -Obtain 3 BPs
- ->1 year corrected gestational age
  - -If BP normal (<90th percentile): no further BPs needed
  - -If BP is high (≥90th percentile): obtain 2 more BPs

Between Patients: Ensure previous patient's blood pressure will not be used for adaptive target
- -Turn machine on and off

Or
- -Clear this history by holding the "history" key for more than 2 seconds

Epic Entry of BPs:
- -First blood pressure can be entered into vitals flow sheet
- -2nd and 3rd blood pressures can be added under "Additional Blood Pressures" tab
  - -This tab is immediately below the "Vitals" tab

If Blood Pressure is High:

- If BP is >99th % in two out of 3 measurements, refer to nephrology
- If ≥90th% but <99th%, recommend repeating measurements in either PCP or specialty care visits
